# Acyclovir‐unresponsive disseminated herpes zoster in B‐cell chronic lymphocytic leukemia

**DOI:** 10.1111/ddg.15870

**Published:** 2025-08-23

**Authors:** Tabael L. Turan, Thomas K. Eigentler, Ulrike Blume‐Peytavi, Kamran Ghoreschi

**Affiliations:** ^1^ Department of Dermatology Venereology and Allergology Charité – Universitätsmedizin Berlin Corporate Member of Freie Universität Berlin Humboldt-Universität zu Berlin, and Berlin Institute of Health Berlin Germany; ^2^ Berlin Institute of Health at Charité – Universitätsmedizin Berlin BIH Biomedical Innovation Academy BIH Charité Junior Clinician Scientist Program Berlin Germany

Dear Editors,

Patients with hematological malignancies are at significantly increased risk of developing herpes zoster (HZ), i.e., reactivation of dormant varicella‐zoster virus (VZV). Potentially fatal HZ‐related complications such as extracutaneous VZV dissemination are more common in these patients.^1^, ^2^ In patients with chronic lymphocytic leukemia (CLL), both the disease itself and the treatment administered are responsible for the dysfunctional immune response. These patients are susceptible to infections and complications, especially within the first few years of diagnosis.^3^, ^4^ Early initiation of potent antiviral agents is prognostically critical in HZ, with intravenous acyclovir (ACV) remaining the treatment of choice.^5^ Resistance of VZV to ACV has been described predominantly in immunocompromised patients, usually conferred by mutations in the viral thymidine kinase (TK) gene.^6^ Second‐line TK‐independent antivirals such as foscarnet (FOS) or cidofovir have sporadically shown efficacy in such cases.^7^, ^8^ Here, we report the case of a patient with B‐cell chronic lymphocytic leukemia (B‐CLL) on watchful waiting, who developed progressive disseminated HZ with pulmonary involvement despite long‐term antiviral therapy including ACV and FOS.

A 60‐year‐old female presented to the emergency department in July 2024 with a two‐day history of a painful vesicular eruption affecting the left V1 dermatome. The patient reported that the rash was preceded by a three‐day period of a localized tingling sensation. The patient reported no other symptoms, such as vision changes, and denied having had a similar rash in the past. She had not previously received HZ vaccine. Past medical history revealed that she had been diagnosed with B‐CLL [del(13q)] in 2013, for which she underwent six cycles of first‐line chemoimmunotherapy with bendamustine and rituximab in 2015/16, followed by watchful waiting. Twenty days prior to admission, a white blood cell (WBC) count of 49.8 × 10^3^/µl and a normal C‐reactive protein (CRP) concentration were measured in blood as part of follow‐up care. There was no hypogammaglobulinemia at that time (IgG, 11.93 g/l).

Upon physical examination, about 25 vesicles were observed outside the primary dermatome, involving the nuchal region, anterior trunk, and upper extremities. No ocular manifestations were noted except for moderate ipsilateral eyelid edema. No abnormal lymph node enlargement was palpable either. Blood sampling showed an elevated CRP level (81.3 mg/l) and relative lymphocytosis (51.3%) with smudge cells (41.2%). Notably, the WBC count (10.4 × 10^3^/µl) was within normal limits, indicating a significant (sub‐)acute decline, as compared to preadmission levels (Figure 1). Peripheral blood flow cytometry confirmed a B‐CLL immunophenotype (CD19 expression > 95%).

**FIGURE 1:**
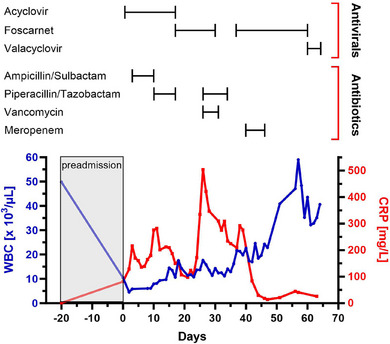
Serum dynamics of C‐reactive protein (CRP; red line) and white blood cells (WBC; blue line) in a hospitalized patient with disseminated zoster and underlying B‐cell chronic lymphocytic leukemia, related to the anti‐infective agents administered.

After clinical diagnosis of HZ V1 with cutaneous dissemination, the patient was started on high‐dose intravenous ACV (10 mg/kg body weight [BW] q8h). As CRP increased to 216.3 mg/l on day 3 of hospitalization without any clinical, laboratory, or radiological signs of an additional infection focus, antibiotic therapy with ampicillin/sulbactam (2 g/1 g q8h) was initiated. Despite an intermediate decline, CRP continued to rise, and antibiotic escalation to piperacillin/tazobactam (4 g/0.5 g q8h) was performed. Thereafter, CRP decreased significantly, and antibiotic therapy was discontinued on day 16 as there was no evidence of bacterial infection. Clinically, however, no response to antiviral therapy was noticed, as new aberrant vesicles spread over large areas of the body, suggestive of ACV‐resistant VZV. On day 14, the vesicular fluid tested highly positive for VZV DNA by polymerase chain reaction. Enzyme immunoassay‐based serology was positive for anti‐VZV IgG (2782 mIU/ml), while anti‐VZV IgM and anti‐VZV IgA were negative.

As of day 16, second‐line treatment with FOS (40 mg/kg BW q8h) was started, administered by intermittent infusion into a peripherally inserted central venous catheter (PICC). Serum CRP decreased by approximately 48% within 5 days of FOS administration, reaching a nadir of 97.7 mg/l on day 21. In parallel, there was minimal clinical improvement of the zosteriform rash. Yet, shortly after, CRP levels > 300 mg/l were measured and the patient's general condition rapidly deteriorated. Following blood culture acquisition, PICC removal, and initiation of empirical piperacillin/tazobactam plus vancomycin (1 g q12h), the patient was transferred to the ICU on day 27 with a blood pressure, pulse rate, oxygen saturation, and body temperature of 138/76 mmHg, 109 bpm, 61%, and 38.5°C, respectively. Computed tomography (CT) of chest on ICU admission showed pulmonary vascular congestion without infiltrates. Microbiology did not yield specific pathogenic microorganisms, entailing cessation of the antibiotic regimen in the further course. Given that all vesicles had dried up by this time, FOS was discontinued after 13 days of treatment. Although the inflammatory parameters displayed a decreasing trend, the patient remained dependent on non‐invasive ventilation. At subsequent bronchoscopy on day 35, VZV DNA was detected in the bronchoalveolar lavage specimen. Furthermore, a follow‐up chest CT revealed extensive bilateral atypical pulmonary infiltrates (Figure 2a,b). Intravenous FOS in combination with meropenem (1 g q8h) was commenced accordingly, resulting in a considerable and sustained decline in CRP. Concurrently, the WBC count returned to preadmission levels. The patient received intravenous immunoglobulin on day 42 in view of a three‐class immunoglobulin deficiency (IgG, 6.28 g/l; IgA, 0.35 g/l; IgM, 0.22 g/l). Finally, we observed a regression of the pulmonary infiltrates on chest X‐ray and an improvement of the patient's respiratory condition. On day 61, FOS was replaced with oral valacyclovir (500 mg q12h) for VZV‐prophylaxis, but nephrotoxicity occurred, necessitating discontinuation of the antiviral medication.

**FIGURE 2:**
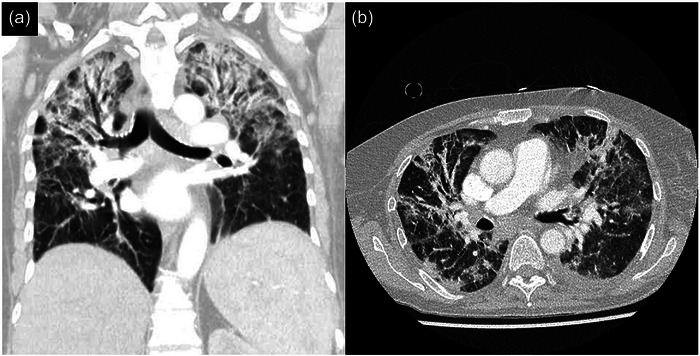
Chest computed tomography with (a) coronal and (b) axial views showing bilateral upper lobe infiltrates on day 38 of hospitalization.

After 65 days of hospitalization, the patient was discharged clinically stable with a follow‐up appointment with the hematologists to evaluate zanubrutinib, a next‐generation Bruton tyrosine kinase inhibitor, as a targeted second‐line treatment for CLL.

Profound immune perturbation contributes to an increased risk of infection in CLL.^9^ Cases of protracted HZ in hematological patients have previously been published.^6^, ^10^ The novelty of this case report is twofold: First, severe HZ occurred in a regularly followed B‐CLL patient on watchful waiting for more than 8 years, i.e., temporally independent of the first diagnosis or initiation of chemotherapy. Second, this is the first case of successful FOS administration in a B‐CLL patient with HZ unresponsive to first‐line ACV treatment.

The seroprevalence > 90% of VZV among unvaccinated immunocompetent subjects places them at high risk of HZ development.^11^ Disseminated HZ with visceral involvement is usually limited to immunocompromised patients, particularly those with active disease or recent initiation of treatment.^12^ In a cohort of CLL patients, VZV reactivation has been reported to occur frequently and up to at least 3 years after first‐line bendamustine plus rituximab, regardless of antiviral prophylaxis.^13^ In addition to the pronounced alterations in T‐cell and neutrophil functioning, hypogammaglobulinemia is considered to be a central factor predisposing CLL patients to infectious complications.^9^, ^14^ Interestingly, none of these risk factors were met in our patient. Thus, VZV reactivation in this case may be interpreted as the initial manifestation of disease progression, indicating the potential need for second‐line treatment in the further course. On a related note, we observed a transient WBC decline during hospitalization, most likely attributable to an increased turnover associated with the acute viral infection.

According to the *German Society for Hematology and Medical Oncology* (DGHO), patients with hematological malignancies require intravenous ACV (10 mg/kg BW q8h) for at least 14 days in case of disseminated HZ.^15^ Resistance of VZV to ACV has been demonstrated in rare cases of immunocompromised patients with AIDS,^16^ hemato‐oncological diseases,^17^ or after hematopoietic stem cell transplantation.^18^ In the present case, there was no sufficient clinical response to the recommended antiviral regimen. Resistance to ACV could have been assessed by phenotypic or genotypic characterization of VZV TK. Genotypic resistance testing in specialized laboratories is preferable for clinical use due to its faster turnaround time. However, mutational VZV analysis is not commonly available in Germany. More importantly, clinical resistance to ACV cannot be verified virologically in many cases, as the specific VZV mutations conferring antiviral resistance have not been comprehensively characterized yet.^17^, ^19^ Besides drug resistance, inadequate ACV dosing might have accounted for clinical treatment failure. For instance, a 6‐year‐old boy with acute lymphocytic leukemia and VZV encephalitis was treated with 20 mg/kg BW of intravenous ACV three times a day and obtained complete functional recovery.^20^ Considering that ACV is mainly renally excreted, dose escalation may be required in patients with a high glomerular filtration rate particularly.

Notably, a biphasic clinical course arose from FOS treatment, with initial complete resolution of the zosteriform rash, followed by severe VZV pneumonia. Temporary discontinuation of FOS after 13 days might have been a decisive factor in this regard, yet this remains speculative, as no virological surveillance has been conducted and the suspected ACV resistance of VZV isolates has not been substantiated genotypically. Nonetheless, prolonged administration of FOS beyond the time when all vesicles have dried up should be considered in future cases of refractory disseminated HZ. Collectively, this rare case of HZ V1 complicated by cutaneous and pulmonary dissemination and clinical ACV resistance provides a valuable reference for the clinical management of patients with B‐CLL.

## CONFLICT OF INTEREST STATEMENT

None.
